# One Dimensional Coordination Polymer of Zn(II) for Developing Multifunctional Nanoparticles

**DOI:** 10.1038/s41598-017-12980-6

**Published:** 2017-10-16

**Authors:** Rashmi A. Agarwal

**Affiliations:** Department of Chemistry, Indian Institute of Technology, Kanpur, 208016 India

## Abstract

A variety of nanoparticles (NPs) including Ag, Au, Pd, Cr and mixed Cu/Fe have been synthesized in a non-activated (without solvent removal) one dimensional coordination polymer (CP) of Zn(II) via two different mechanisms, acid formation and redox activity of the framework. Main driving force to grow these NPs within the cavities of CP is the presence of free oxygens of one of the monodentate carboxylate groups of BDC ligand. These free oxygens act as anchoring sites for the metal ions of the metal precursors. Chemical and physical characteristics of the NPs within the framework have been evaluated by the high resolution transmission electron microscopic (HRTEM) images. Excluding Ag(0) and Pd(0) other NPs are present as combinations of their elemental as well as oxide forms (Au/Au_2_O_3_, Cr/Cr_2_O_3_/CrO_2_ and Cu/Cu_2_O, Fe/FeO). Synthesized Ag NPs within the framework show remarkable antibacterial efficacy at extremely low concentrations. Ag, Au and Cu/Fe NPs show ferromagnetic properties within the framework at room temperature. This polymer has potential to sequester highly toxic Cr(VI) to non toxic Cr(0), Cr(III) and Cr(IV) species.

## Introduction

Coordination polymers with regular channel structures known as porous coordination polymers (PCPs) or metal organic frameworks (MOFs) are excellent materials for promising applications such as separation, gas storage, host-guest chemistry and for catalysis^1–4^. Long range crystalline order of these PCPs/MOFs creates uniform pore size distribution along with tuneable dimensionality and chemical tailoring of the inner surface of the cavities make these structures potential candidate for the fabrication of nanoparticles (NPs). As it is well known that chemical and physical properties of NPs such as thermal and electrical conductivities are different from bulk metals due to more delocalization of free electrons^5^. Free metal NPs have tendency to agglomerate resulting in disappearance of inherent properties of NPs due to high surface energy. It affects long term storage of metal NPs. PCPs/MOFs with controlled size of pores prevent aggregation of NPs due to powerful confinement effect to limit their growth^6^. Immobilization of NPs within the PCPs has been achieved by a variety of methods. Most commonly used method is solution infiltration method where in, desolvated porous MOF is immersed in the metal precursor solution. Due to capillary force, precursor is infiltrated in to the pores followed by reduction with reducing agents^7,8^, or photo catalytic reduction through UV irradiation^9^. In incipient wetness method the volume of solution of metal precursor is same as the total pore volume of the MOF^10^. Double solvent method followed by hydrogen reduction (at 200 °C)^11^, utilizing redox active MOFs without employing reducing agents^12^ chemical vapour deposition of volatile organometallic precursors followed by hydrogenolysis^13^ or thermal decomposition^14^ to reduce the metal precursors or by harnessing photoactive MOF through visible light irradiation^15^ are different approaches to fabricate NPs within the PCPs/MOFs. Recent methods include microwave irradiation^16^, solid grinding followed by hydrogenation^17^ or encapsulation of pre-synthesized NPs in MOFs^18^. At least one requirement is must in all above mentioned methods in the form of heat, radiations, reducing agents, mechanical energy, redox active framework or surfactant, etc. Amongst PCPs redox-active frameworks are useful as they oxidize or reduce certain metal precursors and include them within their structures^19–22^. Redox active Ni(II) based PCPs have been synthesized by Suh *et al*., to grow Ag and Au NPs (~3–4 and ~2 nm respectively). These NPs nucleated initially within the framework while later diffused onto the surface and aggregated with complete or partial dissociation of the framework^19,20^. Most of the NPs have been synthesized within the 2D or 3D structures of the PCPs/MOFs. Herein synthesis of NPs (Ag, Au, Pd, Cr, Cu/Fe) have been carried out in a non-activated 1D nanoporous coordination polymer of Zn(II) at room temperature without utilizing any reducing agent. First time Cr NPs have been synthesized by redox active reaction between coordinated Zn(II) metal ions of the framework and strongly oxidising Cr(VI) ions of the metal precursor proved by EPR spectrum. Other NPs were grown within the cavities of PCP due to acid formation (HNO_3_/HCl). Solvent-accessible void volume of this polymer is ~7.7% and BET surface area is 3.896 m²/g. After synthesis of NPs, integrity of the framework is maintained. These NPs integrated structures are characterized as **Ag@Zn-PCP**, **Au/Au**_**2**_ **O**_**3**_ **@Zn-PCP**, **Pd@Zn-PCP**, **Cr/Cr**_**2**_ **O**_**3**_ **/CrO**_**2**_ **@Zn-PCP** and **Cu/Cu**_**2**_ **O,Fe/FeO@Zn-PCP**.

Ag NPs shows excellent antibacterial properties by directly using **Ag@Zn-PCP** in significantly small quantity. **Ag@Zn-PCP** and **Au/Au**_**2**_ **O**_**3**_ **@Zn-PCP** exhibit ferromagnetism while **Cu/Cu**_**2**_ **O**, **Fe/FeO@Zn-PCP** acts as a soft ferromagnet at room temperature. The host template **Zn-PCP** has the potential to sequester highly toxic Cr(VI) to non toxic Cr(0), Cr(III) and Cr(IV) species. It is also a good candidate to integrate NPs from mixed metal precursors (Cu/Fe).

## Results

**Figure 1:**
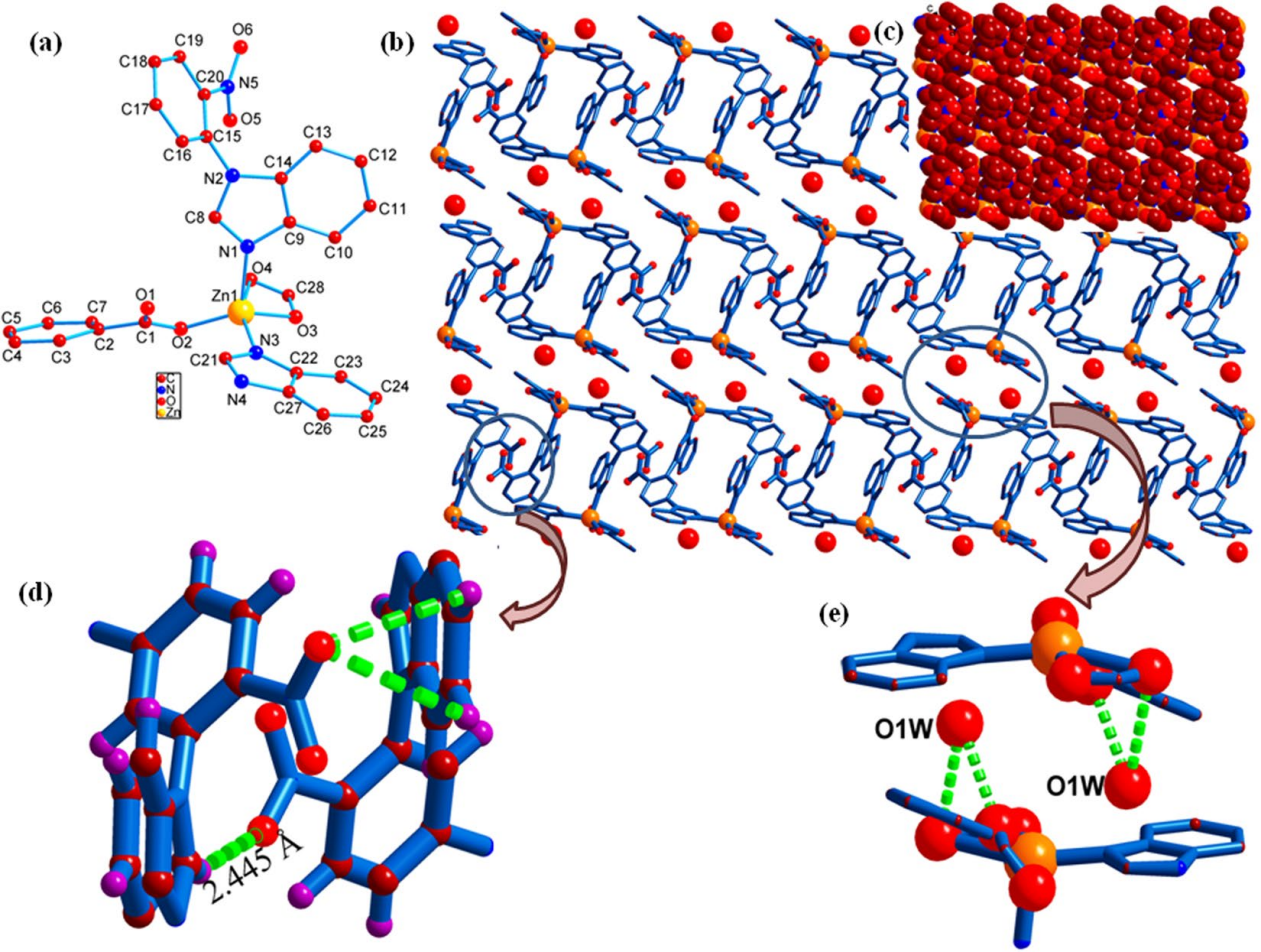
Crystal strucrure of Zn-PCP. (**a**) Coordination environment around metal centre, (**b**) packing of 1D ladders in to 3D structure. **(c)** Space filled diagram. **(d)** and **(e)** Hydrogen bonding interactions between two adjacent ladders.

A number of NPs have been integrated by utilizing a previously reported non-activated 1D coordination polymer {[Zn(NPBI)(BDC)]∙H_2_O}_*n*_ [NPBI = 1,1′-(4-nitro-1,3-phenylene)bis(1H-benzo[d]imidazole), BDC = 1,3-benzenedicarboxylic acid]^23^ at room temperature without using reducing agent. In this polymer Zn(II) adopts square pyramidal ZnN_2_O_3_ geometry coordinated by two benzimidazole nitrogen atoms of two NPBI ligand and two carboxylate groups of two BDC^2−^ one in monodentate and second one in bidentate fashion (Fig. 1a) constructing a one dimensional ladder. Strong hydrogen bonding interactions between ladders as shown in the Fig. 1d and e generate a 3D supramolecular architecture consisting of 1D dumbbell shaped cavities of diameter ~3.2 Å (considering van der Waals radii) (Fig. 1b–c).

Due to monodentate binding mode of one carboxylate of BDC^2−^ at the inner surface of the cavities makes this PCP a suitable candidate for the synthesis of NPs. Because these free oxygens of carboxylate groups act as anchoring sites for the metal ions of the metal precursors.

To nucleate and grow Ag, Au, Pd, Cr and Cu/Fe NPs, host template (**Zn-PCP**) was immersed in a methanol/water solution of metal precursors (AgNO_3_, HAuCl_4_, PdCl_2_, K_2_CrO_4_ and CuCl_2_ · 2H_2_O/Fe(NO_3_)_3_ · 9H_2_O) for 48 h at room temperature under stirring. Yellow colour of the **Zn-PCP** of all five different solutions were slightly changed just after 10 minutes indicating interactions have been started between host PCP and metal precursors. Reactions were allowed for 48 h to check the probable mechanism because of the different rate of anchoring of metal precursors with free oxygens of monodentate carboxylate groups of BDC^2−^.

**Figure 2:**
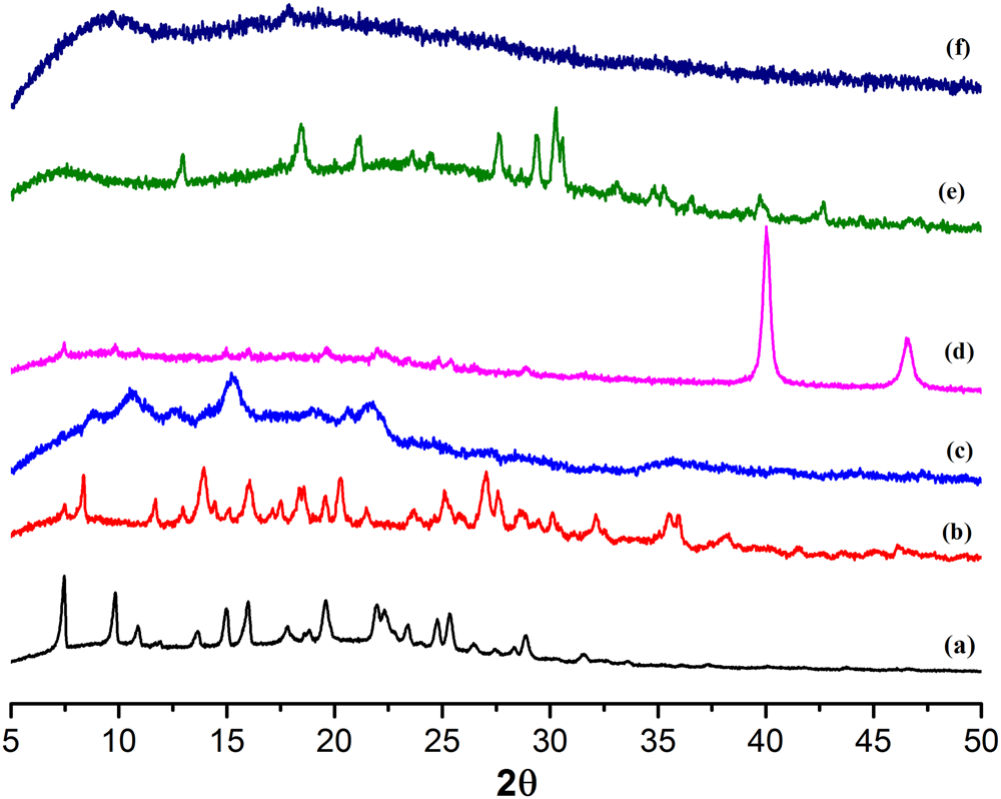
PXRD patterns. (**a**) Host template (Zn-PCP). (**b**) Ag@Zn-PCP. (**c**) Au/Au_2_O_3_@Zn-PCP. (**d**) Pd@Zn-PCP. (**e**) Cr/Cr_2_O_3_/CrO_2_@Zn-PCP. (**f**) Cu/Cu_2_O,Fe/FeO@Zn-PCP.

Room temperature PXRD peak at 2θ = 38.16° (Fig. 2b) can be assigned to 111 reflection of the face centered cubic (fcc) structure of the metallic Ag(0) with JCPDS file number 04–0783 according to literature reports^24^. In case of Au integrated framework, the nanoparticles are too small (2–5 nm) that peaks for these NPs are not visible (Fig. 2c). High intensity XRD peaks at 40° and 46.6° (Fig. 2d) are attributed to the face centered cubic metallic Pd in 111 plane^25^. A peak position for Cr(0) at 2θ = 44.3° corresponds to 110 reflection plane with JCPDS number 06–0694^26^. Other diffraction peaks at 33° (104), 36.5° (110), 39.7° (006) are ascribed to Cr_2_O_3_ NPs while a peak at 30.1° (110) corresponds to the CrO_2_ NPs (Fig. 2e). A large stress is observed in case of mixed NPs (Cu/Fe) PXRD spectrum as shown in Fig. 2f that may be due to the competition between two different metal ions of different metal precursors or due to heterogeneous environment. Due to high flexibility of the host PCP, metal peaks of the host in the PXRD spectra of Fig. 2c, d and f are shifted due to high or large size growth of the metal NPs.

EDS (Figs S1 and S2) and XPS data show that both the metallic species (Zn(II) metal ions of the framework and metal NPs) coexist in the framework. Chemical nature of different NPs has been characterized by XPS spectra (Fig. S3). The spectrum for **Ag@Zn-PCP** (Fig. S3b) shows the characteristic binding energies at 366.4 and 372.8 eV related to Ag 3d_5/2_ and Ag 3d_3/2_ respectively^27^ while for the **Au/Au**_**2**_ **O**_**3**_ **@Zn-PCP** exhibits two peaks at 82.4 and 85.6 eV corresponding to 4 f binding energies of Au(0). In this case size of the Au NPs is small (2–5 nm) therefore their binding energy decreases as recently pointed out by Dalascu *et al*.^28^. At higher resolution, the second peak is deconvoluted in to two peaks (Fig. S4a) which is attributed to Au(III)^29^. XPS spectrum of **Pd@Zn-PCP** shows binding energies at 335.2 and 340 eV (Fig. S4b) correspond to Pd(0)^30^. For Cr NPs integrated framework (Fig. S5a) binding energies at 575.2, 576.8 and 578.4 eV are ascribed to Cr(0), Cr_2_O_3_ and CrO_2_ respectively^31^. XPS of mixed nanoparticles (Cu/Fe) shows binding energy at 931.2 eV which corresponds to Cu or Cu_2_O because they cannot be identified by simply deconvolution due to very close binding energy^32^. But this problem is resolved from the position of their LMM-2 auger transition in XPS spectra for Cu and Cu_2_O at 568.8 and 570.4 eV respectively^33^, while XPS peaks at 708.8 and 711.2 eV are due to the presence of Fe and FeO (Fig. S5b and c)^34^.

EPR spectra of NPs (Ag, Pd, Cu/Fe) integrated frameworks (Fig. S6) show that there is single sharp peak at g = 2.06–1.99 for NPs formation. But in case of Au NPs growth two EPR peaks (Fig. S6b) can be seen at g = 2.02 and 1.99 revealing that there is heterogeneous distribution of NPs with two different size range. EPR spectrum of **Cr/Cr**_**2**_ **O**_**3**_ **/CrO**_**2**_ **@Zn-PCP** shows one peak for Zn(III) at g = 4.35 which is oxidised state of Zn(II) as in the literature one peak for Fe(III) is reported at g = 4.3 due to quantum mechanical mixed states^35^. It is really difficult for Zn(II) to stay in Zn(III) oxidised state according to previous studies^36–38^ but in this framework Zn(II) is coordinated so it can sustain this oxidised state due to strong oxidising nature of Cr(VI) in acidic media because hydroxide anions combine with the K cations of the K_2_CrO_4_ forming KOH in aqueous solution. Another peak is present at g = 2.06–1.88 for NPs formation with broad size distribution showing redox reaction for the NPs synthesis.

**Figure 3:**
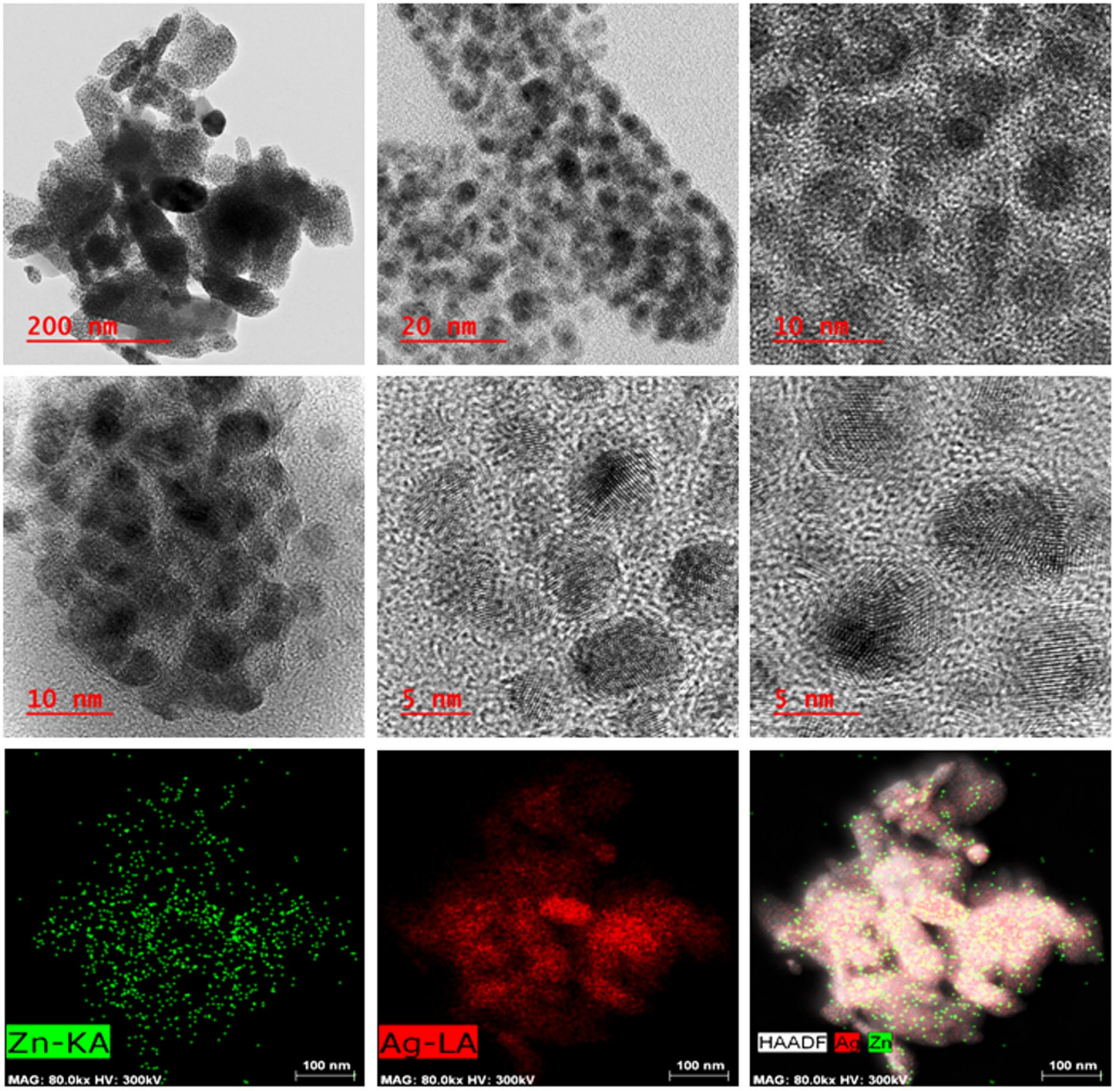
HRTEM and HAADF-STEM images of Ag@Zn-PCP showing that Ag NPs are present within the polymer with clearly visible lattice fringes.

**Figure 4:**
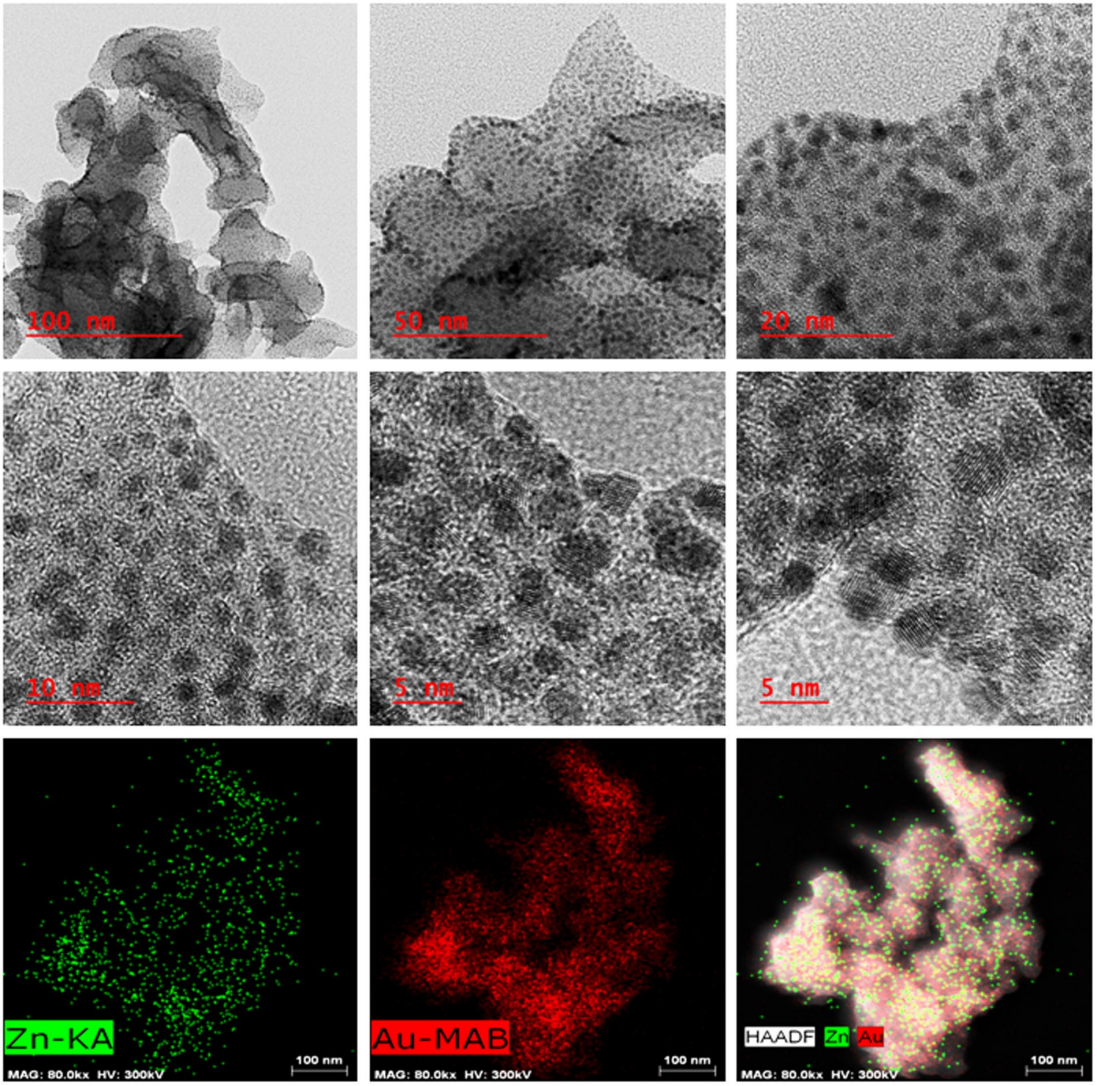
HRTEM and HAADF-STEM images of Au/Au_2_O_3_@Zn-PCP with clearly visible lattice fringes of Au/Au_2_O_3_.

HRTEM images (Figs 3 and 4, Figs S7–9) show that all NPs are spheroids except Pd NPs which are of different shapes including truncated, triangular, cuboid and spheroid with the size range of 30–70 nm. Ag and Au/Au_2_O_3_ NPs have small diameter of 5–10 and 2–5 nm respectively while Cr/Cr_2_O_3_/CrO_2_ NPs have heterogeneous distribution (3–6 nm) and Cu/Cu_2_O,Fe/FeO NPs have size range of 3–4 nm. Lattice fringes of synthesized NPs are clearly visible in all HRTEM images and their size is much larger than cavity size (0.32 nm). HAADF-STEM images provide elemental composition and crystal information at atomic scale (Figs 3 and 4, Figs S7–9).

TGA analysis shows reduced stability of NPs integrated frameworks than the host framework over a temperature range as shown in Fig. S10. This is ascribed to high reactivity of the NPs due to large surface area to volume ratio within the cavities of the host template.

## Discussion

NPs have been synthesized via two different mechanisms as shown by EPR peaks and confirmed by FTIR spectra. Acid formation (HNO_3_/HCl)^39^ takes place to grow Ag, Au, Pd and mixed Cu/Fe NPs. Formation of NPs by acid formation is not very common phenomenon. But in the literature it is mentioned that HNO_3_ is one of the products via redox reaction^40^. During reaction solvated metal precursor enters in the cavities of host template where these metal ions get anchor with the free oxygens of the monodentate carboxylate groups of the BDC^2−^ linker. As it is well known that Ag(I) and Pd(II) ions are positively charged species. These species will be attracted by hydrophilic carboxylate ions to anchor positively charged metal ions^41^. Cr(VI) has high tendency to bind with oxygen as well as Au(III) also has binding properties with the free oxygens of the carboxylate groups^42,43^. It is clear from FTIR spectra (Fig. S11) that C=O stretching of the host framework at 1620 cm^−1^ shifts to a lower wave number at 1606–1609 cm^−1^ due to anchoring of metal ions. Anchored metal ions get reduced to metal NPs with simultaneous formation of HNO_3_ (for metal precursors which are present in the form of metal nitrates) or HCl (if metal precursors are in the form of metal chlorides). Peak splitting of 1351 cm^−1^ of the **Zn-PCP** in to ~1337 cm^−1^ and 1376–1385 cm^−1^ shows presence of HNO_3_ in NPs integrated frameworks (Fig. S11). OH stretching of the water molecule is shifted towards higher wave number (Fig. S12) for those NPs integrated frameworks where HCl is produced through redox reaction for NPs synthesis as shown in IR spectra^44^. Cr NPs have been synthesized through redox active reaction between coordinated metal ions Zn(II) and Cr(VI) ions of the metal precursor. It happens due to strong oxidising nature of Cr(VI), which oxidises framework metal ion Zn(II) to Zn(III) and itself get reduced forming Cr NPs. To neutralize this extra positive charge of Zn(III) ion OH^−^ anion are formed through dissociation of water solvent molecules. This O-H stretching peak is narrow in Cr NPs encapsulated framework confirming presence of hydroxide anion (Fig. S13). Due to large ionic radii of this ion framework is stretched which can be seen in IR spectra.

Due to high flexible nature of the framework even at the room temperature^23^, agglomeration of the NPs takes place leading the particle size of metal NPs which are far above the cavity size of PCP.

To check whether methanol acts as a reducing agent, reactions have been performed only in the presence of water medium but all the products have been obtained. It confirms that methanol does not participate to reduce metal ions of the metal precursors.

**Figure 5:**
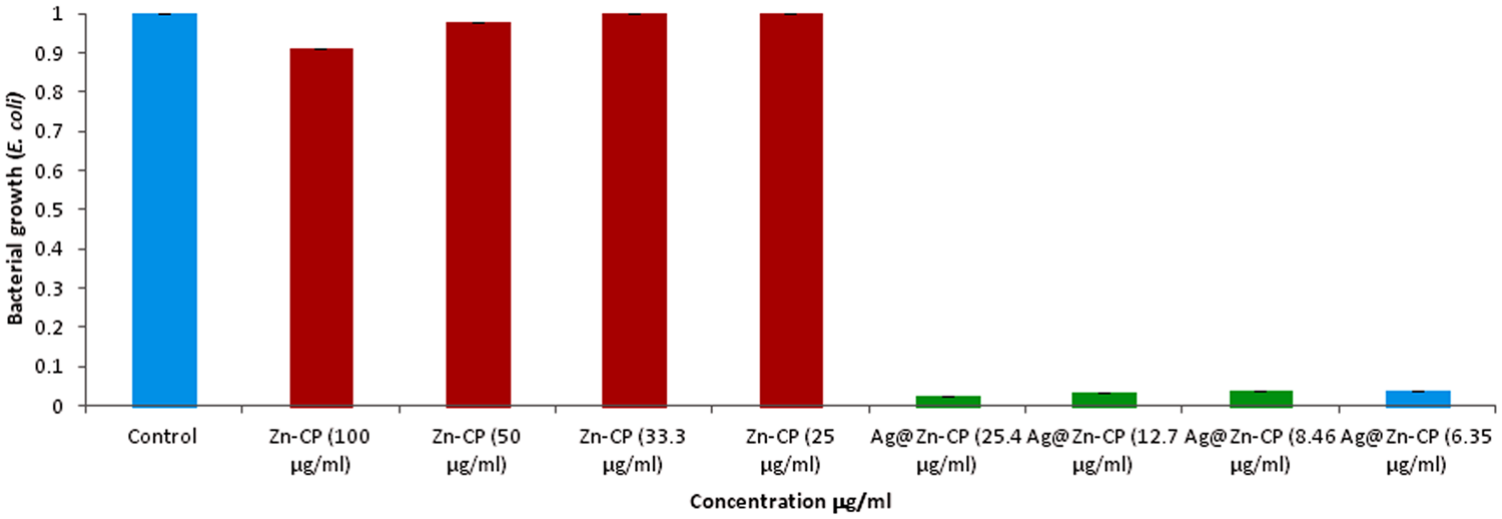
*E. coli* bacterium growth inhibition by Ag@Zn-PCP.

Host framework does not show any efficacy towards growth inhibition of *E. coli* (Gram-negative) whereas Ag NPs integrated framework shows higher antibacterial effect at significantly low concentrations. As it is clear from Fig. 5 that decreasing the concentration of **Ag@Zn-PCP** from 25.4–6.35 µg/ml (~6.35–1.58 µg/ml Ag NPs concentration) ~98% bacterial growth is inhibited. Actual concentrations of the extracted Ag NPs will be lower than the calculated values revealing excellent antibacterial properties. It is known that growth inhibition activity of AgNPs increases with decreasing their size^45–47^. In this case size of Ag NPs is very small (5–10 nm) that is attributed to excellent antibacterial efficacy. It shows potential for biomedical applications.

It may be possible that in case of Gram-positive bacteria due to presence of thick multilayered peptidoglycan layer these Ag NPs may not work efficiently as reported by recent study^48^.

**Figure 6:**
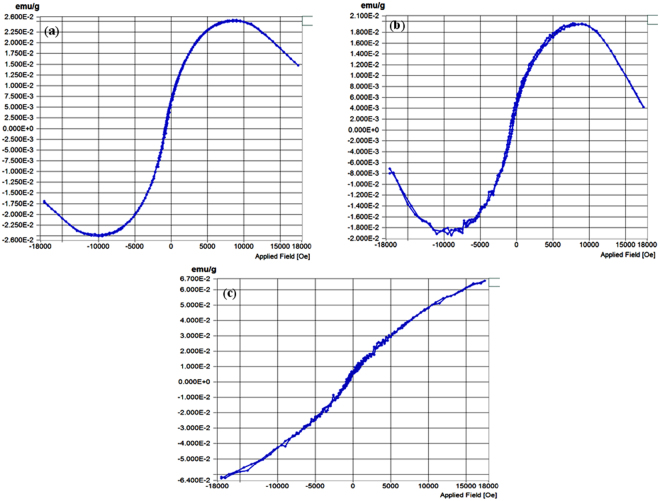
Magnetization curves at room temperature. (**a**) Ag@Zn-PCP. (**b**) Au/Au_2_O_3_@Zn-PCP. (**c**) Cu/Cu_2_O, Fe/FeO@Zn-PCP.

Mass magnetization curves (Fig. 6) of **Ag@Zn-PCP** and **Au/Au**_**2**_ **O**_**3**_ **@Zn-PCP** show that both have magnetism in ground state without applying magnetic field at 300 K. After application of magnetic field at room temperature coercive field (Hc) values are 79 and 145 Oe while remnant magnetization (Mr) values are 0.000061 and 0.00068 emu/g respectively. At room temperature Hc and Mr are not zero showing strong ferromagnetism for these NPs integrated frameworks with their saturation magnetization (Ms) of ferromagnetic signal at 0.025 and 0.019 emu/g respectively. Magnetic curve of mixed NPs integrated framework (**Cu/Cu**_**2**_ **O,Fe/FeO@Zn-PCP**) exhibits soft ferromagnetism at room temperature with Hc, Mr and Ms values 120 Oe, 0.000195 and 0.064 emu/g. Since this magnetic characterization data have been taken on NPs integrated frameworks, if these NPs were free from their membranes then the magnetic values would be definitely higher because these polymeric membranes act as a non-magnetic barrier^49^.

**Figure 7:**
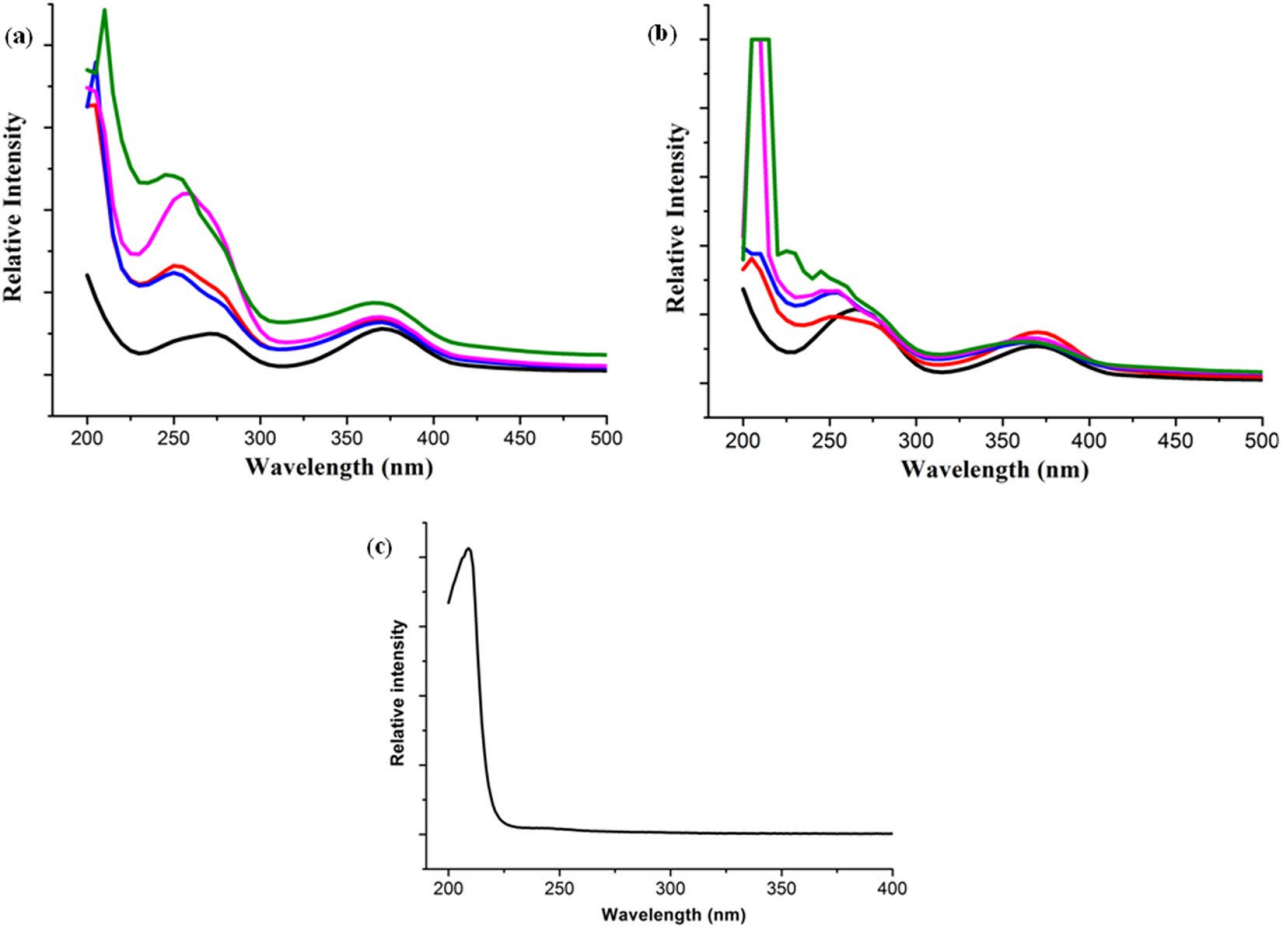
UV-vis spectra. (**a)** Black line for 1000 ppm aqueous solution of K_2_Cr_2_O_7_; red line for 1 h; blue line for 2 h; pink line for 4 h; green line for 10 h treatment with **Zn-PCP**. **(b)** Black line for 2000 ppm aqueous solution of K_2_Cr_2_O_7_; red line for 1 h; blue line for 2 h; pink line for 4 h; green line for 10 h treatment with **Zn-PCP**. **(c)** Absorbance of 1000 ppm aqueous solution of KOH.

As it has been discussed earlier that **Zn-PCP** template can reduce Cr(VI) to non toxic species Cr(0), Cr(III) and Cr(IV) effectively. It has been shown by UV-vis spectra of K_2_Cr_2_O_7_ solutions (1000 and 2000 ppm) treated with **Zn-PCP** for different time intervals (1–12 h) (Fig. 7a and b). The UV-vis spectrum of aqueous solution of K_2_Cr_2_O_7_ exhibits two principal bands falling around 370 and 265 nm^50^. These bands shift towards lower wavelength with the formation of a third new peak at 205–210 nm (Fig. 7c) which is attributed to the KOH. This peak intensity increases with treatment time due to more concentration of KOH in the solution with the nucleation and growth of NPs within the cavities of PCP. As absorption of Cr_2_O_7_^−2^ is shifting towards lower wavelengths with increasing treatment time infers that aqueous solutions of K_2_Cr_2_O_7_ undergo dilution with time. The mechanism for the synthesis of Cr NPs involves reduction of Cr(VI) and oxidation of coordinated metal ion Zn(II) of the **Zn-PCP** through redox reaction due to strong oxidising capability of Cr(VI).

In conclusion a simple approach for the synthesis of multifunctional NPs (Ag, Au/Au_2_O_3_, Pd, Cr/Cr_2_O_3_/CrO_2_ and Cu/Cu_2_O,Fe/FeO) at room temperature has been described by utilizing a 1D coordination polymer of Zn without using any reducing agent and surfactant. Excellent antibacterial properties have been shown by **Ag@Zn-PCP** at significantly low concentrations. This Ag NPs integrated PCP as well as **Au/Au**_**2**_ **O**_**3**_ **@Zn-PCP** shows ferromagnetism at room temperature while mixed NPs (Cu/Cu_2_O, Fe/FeO) integrated framework acts as a soft ferromagnet. If these NPs were free from the host framework then their magnetic properties would have been definitely improved because this host framework acts as non-magnetic barrier. Host template is also capable to reduce highly toxic Cr(VI) to their non toxic Cr NPs. Synthesis of other functional NPs within this host framework are in progress.

## Experimental Section

### Materials

All chemicals and solvents were of reagent grade and used without further purification for the synthesis.

### Physical measurements

Powder X-ray diffraction (PXRD) was performed using a Rigaku Rint 2000 X-ray diffractometer with CuKα radiation. X-ray photoelectron spectra were measured on a Sigma Probe. Fourier transform infrared spectra (FTIR) were obtained (KBr disk, 400–4000 cm^−1^) using a Perkin-Elmer model 1320 spectrometer. HRTEM images were obtained on a FEI Titan G2 60–300. EPR spectra were recorded on a Bruker EPR EMX spectrometer. Thermo-gravimetric analyses were recorded using a Mettler Toledo (heating rate of 10◦C/min) TGA instrument. Absorbance was determined by using UV/Vis spectrophotometer Varian Cary 100. The magnetic properties were analyzed on a LDJ9600 vibrating sample magnetometer.

**Zn-PCP** {[Zn(NPBI)(BDC)]∙H_2_O}_*n*_ was synthesized as previous reports^23^.

### Synthesis of Ag@Zn-PCP

Host framework **Zn-PCP** (0.03 g, 0.050 mmol) was immersed in a methanol/water (3 ml, 2:1 v/v) solution of AgNO_3_ (0.025 g, 0.15 mmol) at room temperature for 48 h under stirring. A brown coloured solid was filtered and washed several times with excess of methanol to remove free AgNO_3_. This solid residue was dried under vacuum comprising of 25 wt% **Ag@Zn-PCP** (the mass ratio of Ag to **Zn-PCP** was 25%).

### Synthesis of Au/Au_2_O_3_@Zn-PCP, Pd@Zn-PCP, Cr/Cr_2_O_3_/CrO_2_@Zn-PCP and Cu/Cu_2_O,Fe/FeO@Zn-PCP

Same procedure was followed except that in place of AgNO_3_, HAuCl_4_ (0.029 g, 0.15 mmol), PdCl_2_ (0.026 g, 0.15 mmol), K_2_CrO_4_ (0.029 g, 0.15 mmol) and a mixure of Fe(NO_3_)_3_ · 9H_2_O (0.60 g, 0.15 mmol), CuCl_2_ · 2H_2_O (0.025 g, 0.13 mmol) were used as metal precursors. After drying observed wt% were 21, 15, 5 and 3 respectively.

### Cell culture and treatment

*Escherichia coli* (*E. coli* DH5α) strain was used for drug sensitivity assay. *E. coli* strains were stored in Luria-Bertani (LB) medium containing 15% glycerol at −80 °C, and were grown in LB broth at 37 °C for 16 h with shaking at 250 rpm. *E. coli* DH5α were assessed for their cell growth by measuring their turbidity at 600 nm (OD600). OD > 2.5 at 600 nm was checked for sufficient growth and further drug treatment. Bacterial cultures (OD600 > 2.5) were sub-cultured in LB broth with different concentrations of sample named **Ag@Zn-PCP** and then incubated at 37 °C for 16 h with shaking at 250 rpm. OD values were taken after this step. Triplicate samples from each treatment were obtained for the determination of mean values and standard deviations.

## Electronic supplementary material

Supplementary Information
