# Supplementary material for: One Dimensional Coordination Polymer of Zn(II) for Developing Multifunctional Nanoparticles
Source: Sci Rep. 2017 Oct 16;7:13212. doi: 10.1038/s41598-017-12980-6 (PMC5643562; doi:10.1038/s41598-017-12980-6)
Supplement: Supplementary file 1 — Supplementary Information [file 41598_2017_12980_MOESM1_ESM.pdf]

One Dimensional Coordination Polymer of Zn(II) for Developing Multifunctional Nanoparticles

Rashmi A. Agarwal*

Department of Chemistry, Indian Institute of Technology Kanpur, 208016, India

Supplementary Information

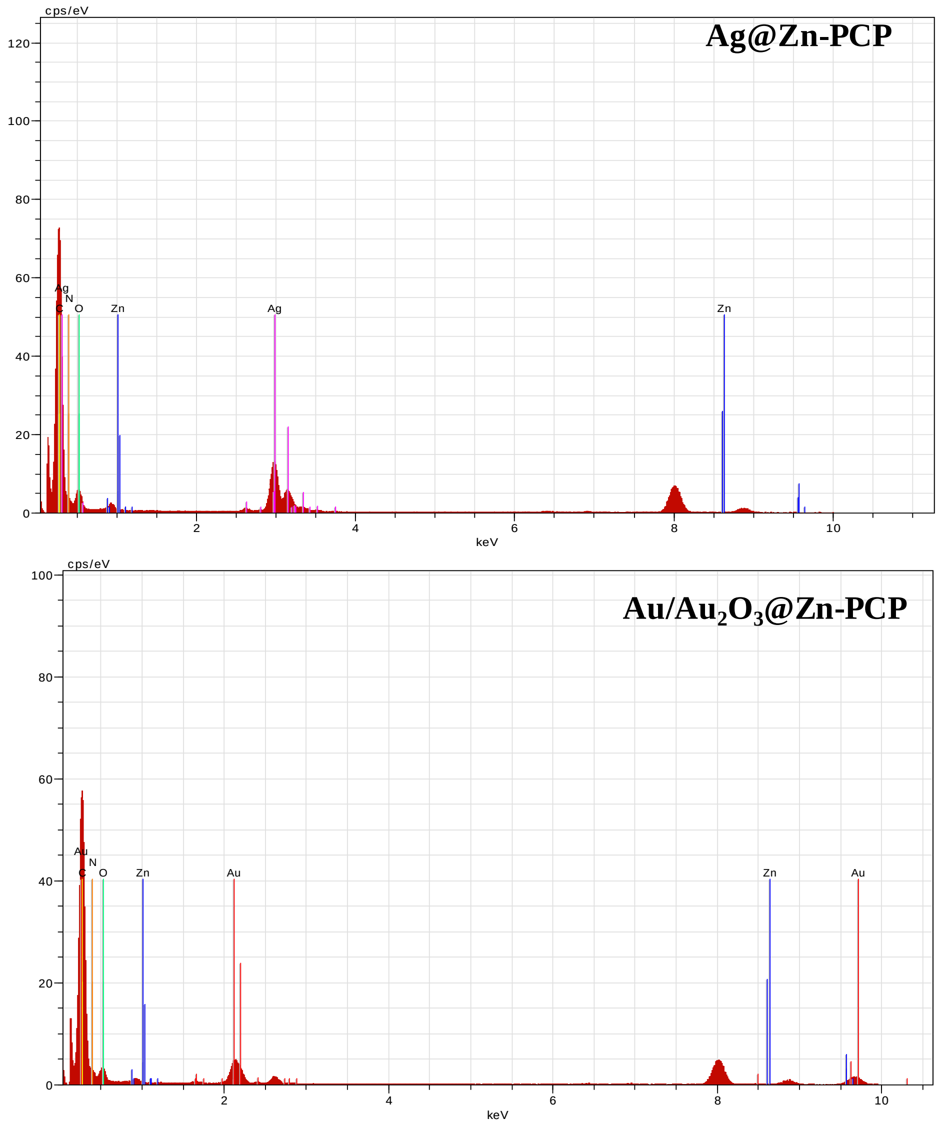

Figure S1. Energy dispersive spectroscopic data of Ag and Au NPs integrated frameworks.

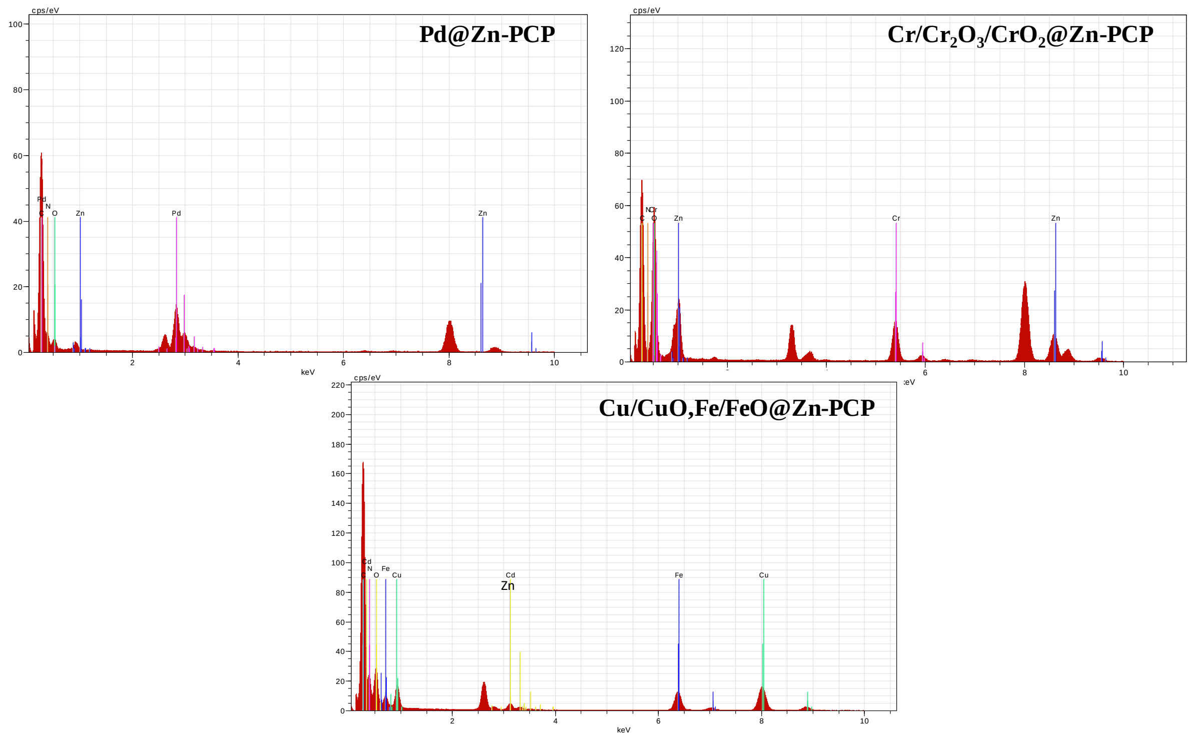

Figure S2. Energy dispersive spectroscopic data of Pd, Cr and Cu/Fe NPs integrated frameworks.

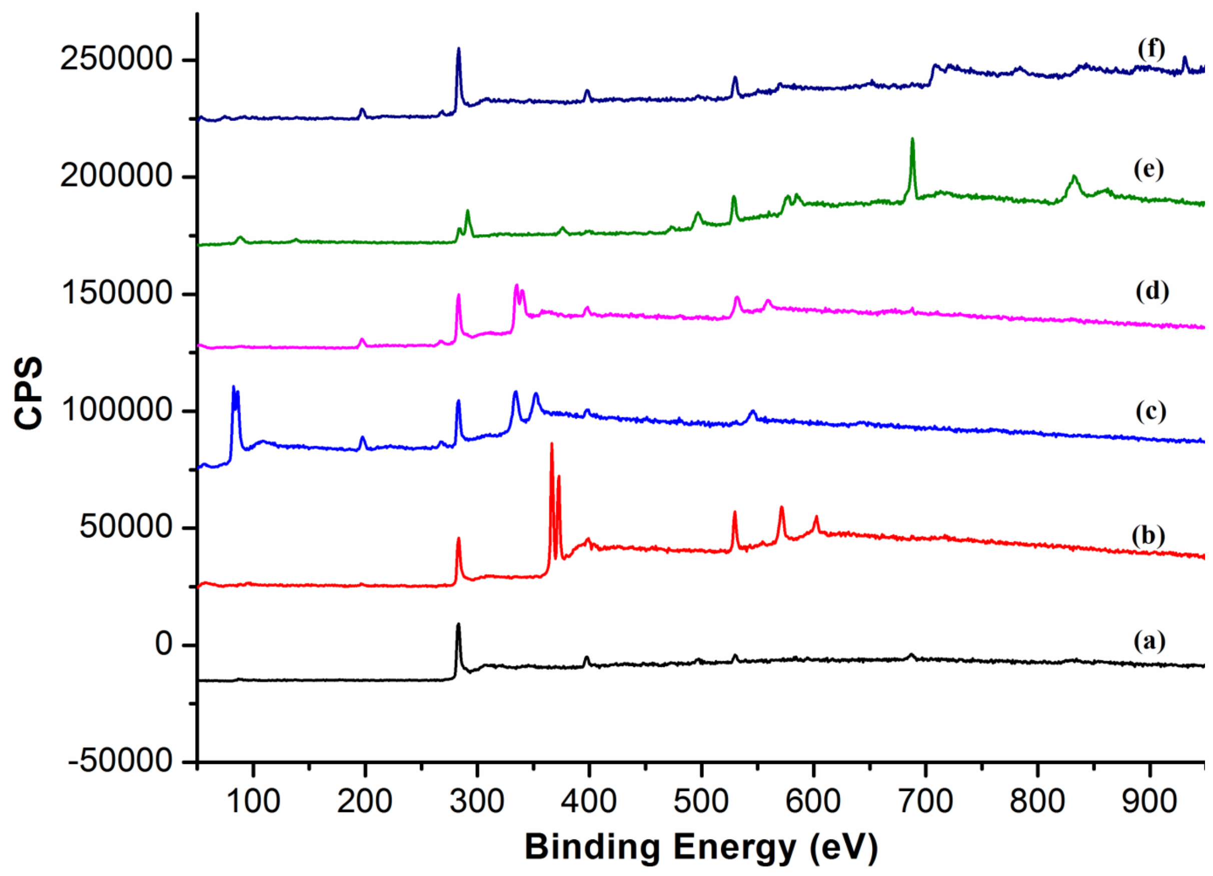

Figure S3. X-ray photoelectron spectra. (a) Zn-PCP. (b) Ag@Zn-PCP. (c) Au/Au_2O_3@Zn-PCP. (d) Pd@Zn-PCP. (e) Cr/Cr_2O_3/CrO_2@Zn-PCP. (f) Cu/CuO,Fe/FeO@Zn-PCP.

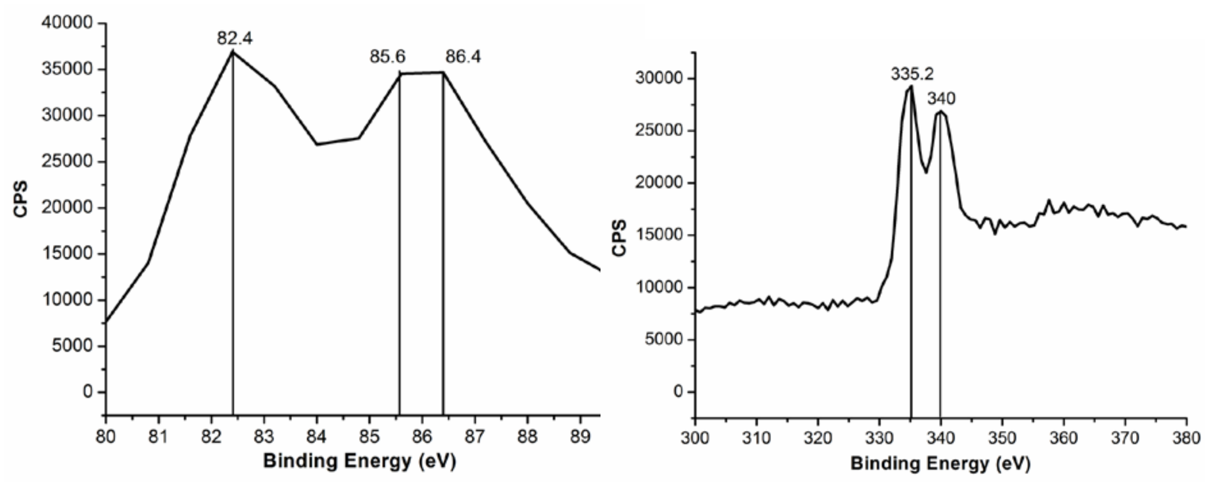

(a) (b)

Figure S4. High resolution X-ray photoelectron spectra. (a) Au/Au_2O_3@Zn-PCP. (b) Pd@Zn-PCP.

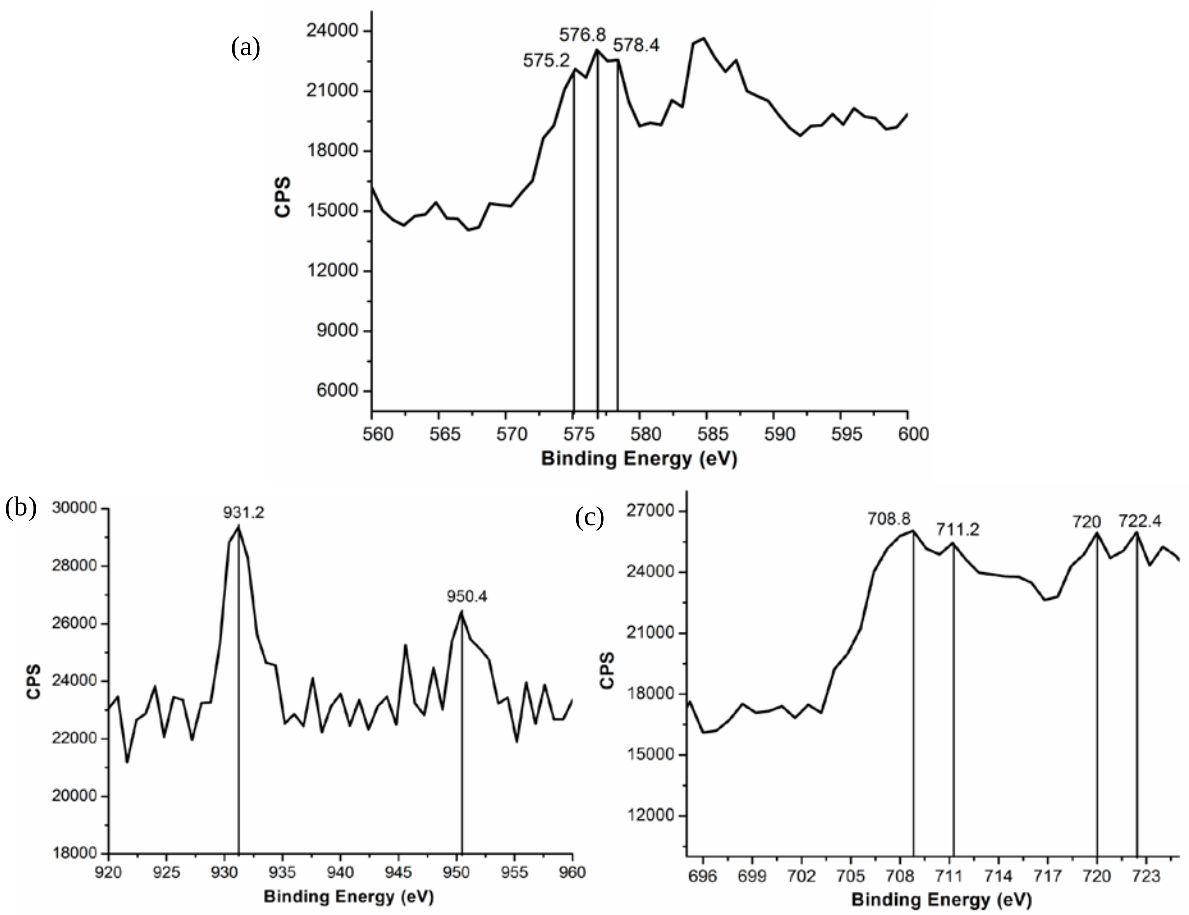

Figure S5. High resolution X-ray photoelectron spectra. (a) Cr/Cr_2O_3/CrO_2@Zn-PCP. (b), (c) Cu/Cu_2O,Fe/FeO@Zn-PCP.

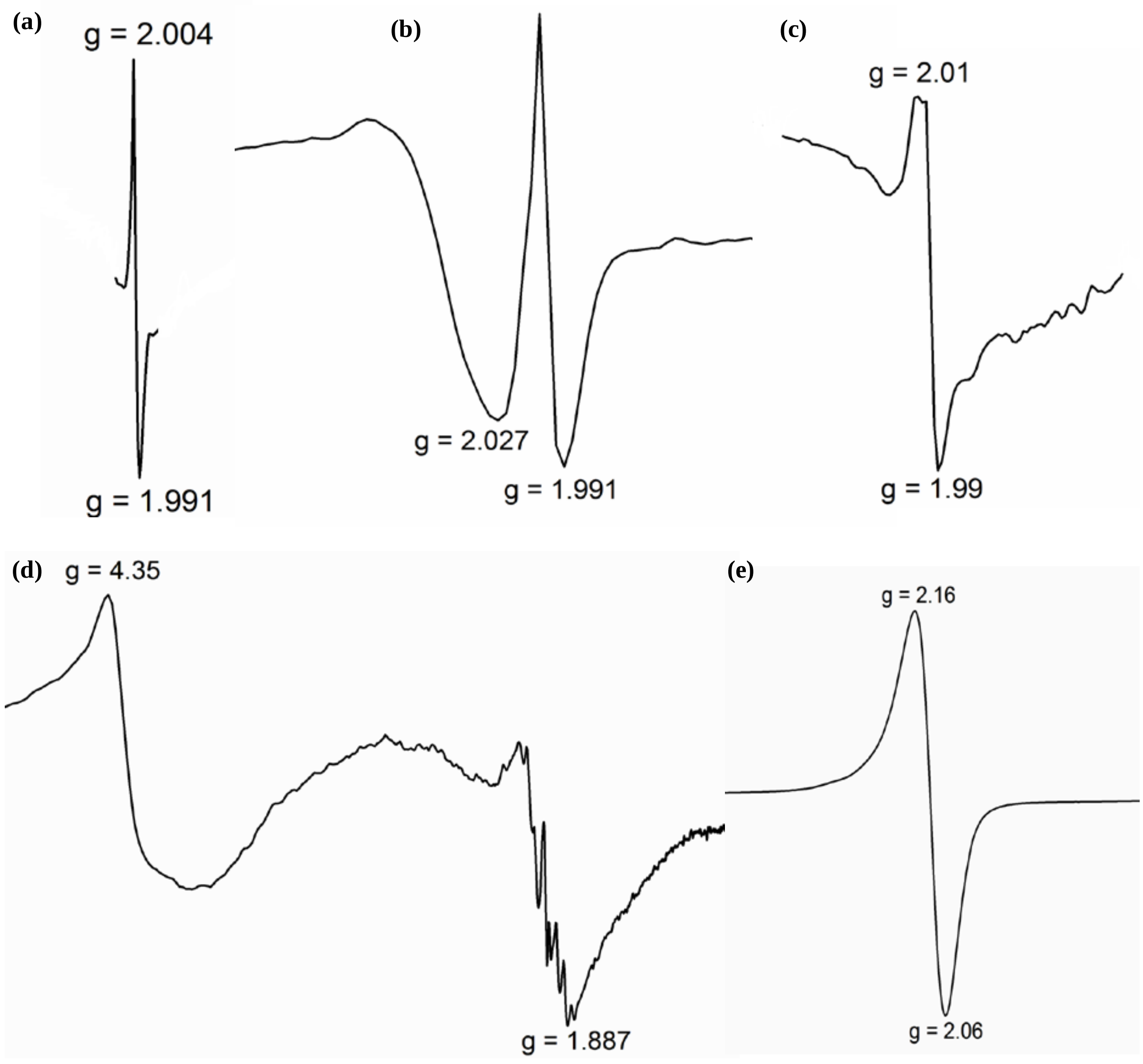

Figure S6. EPR spectrum. (a) Ag@Zn-PCP. **(b) Au/Au_2O_3@Zn-PCP**. **(c) Pd@Zn-PCP**. **(d) Cr/Cr_2O_3/CrO_2/CrO_3@Zn-PCP**. (e) **Cu/Cu_2O,Fe/FeO@Zn-PCP**.

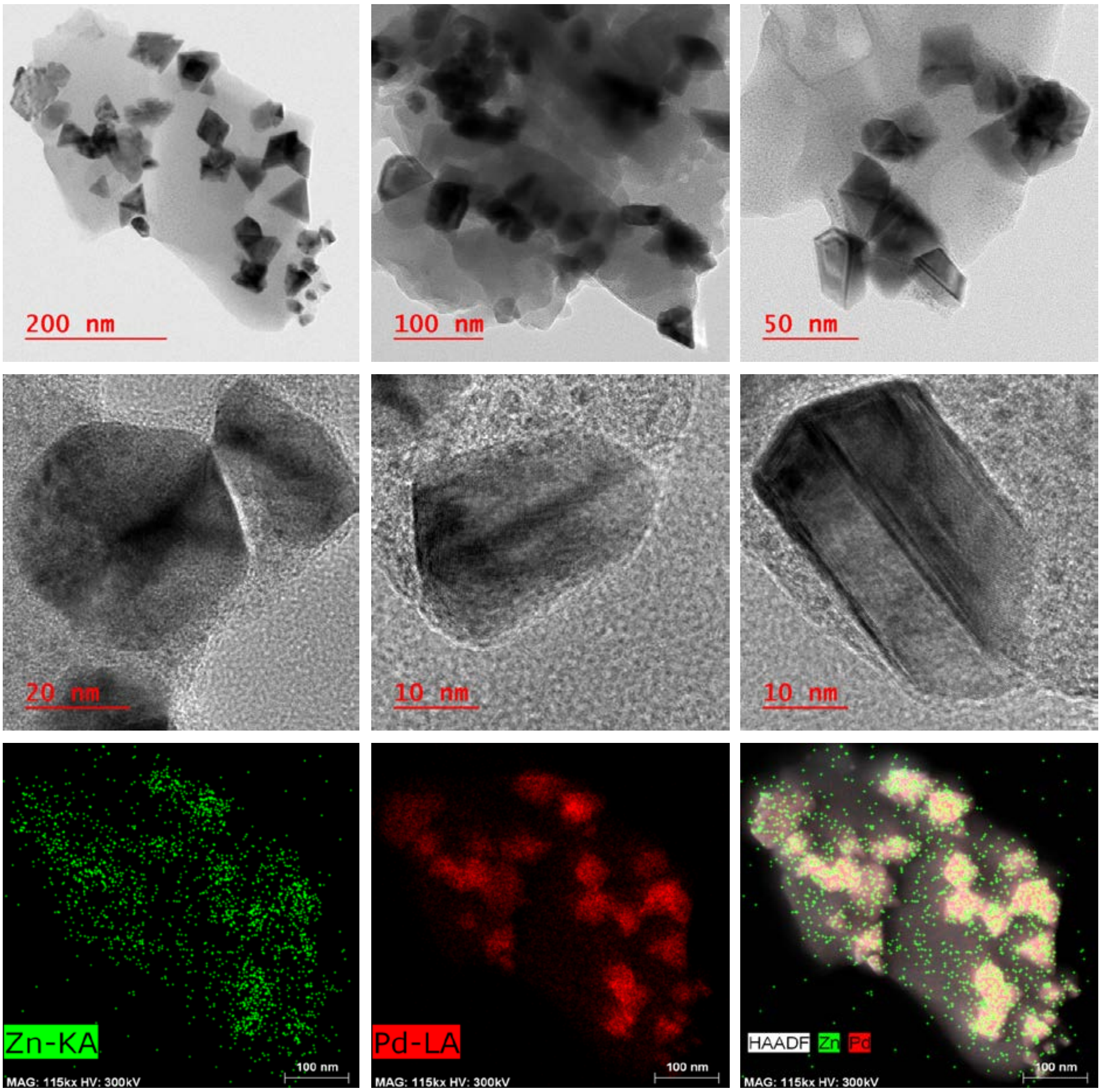

Figure S7. HRTEM and HAADF-STEM images of Pd@Zn-PCP.

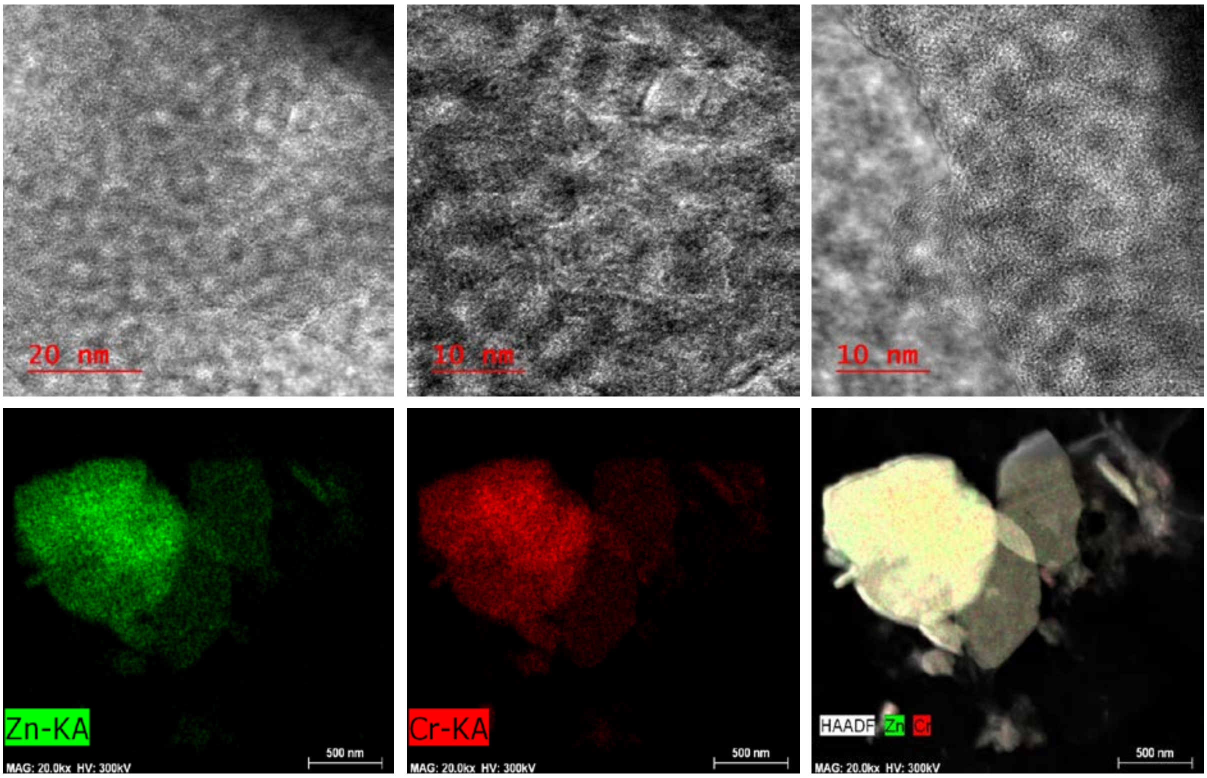

Figure S8. HRTEM and HAADF-STEM images of $Cr/Cr_2O_3/CrO_2$@Zn-PCP.

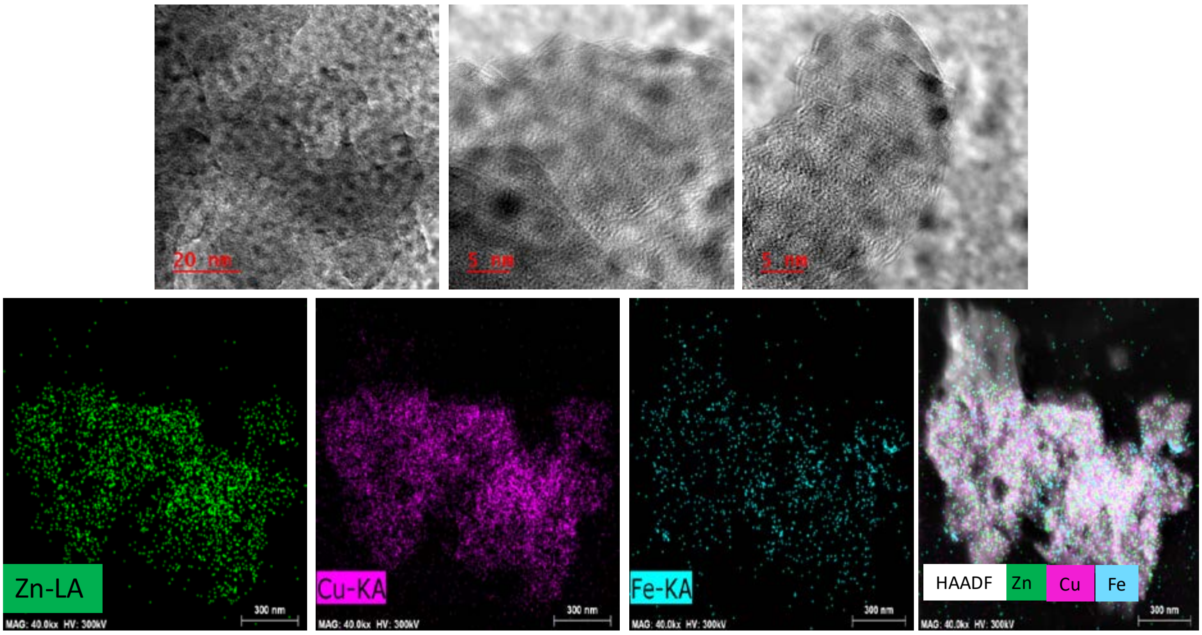

Figure S9. HRTEM and HAADF-STEM images of Cu/Cu_2O,Fe/FeO@Zn-PCP.

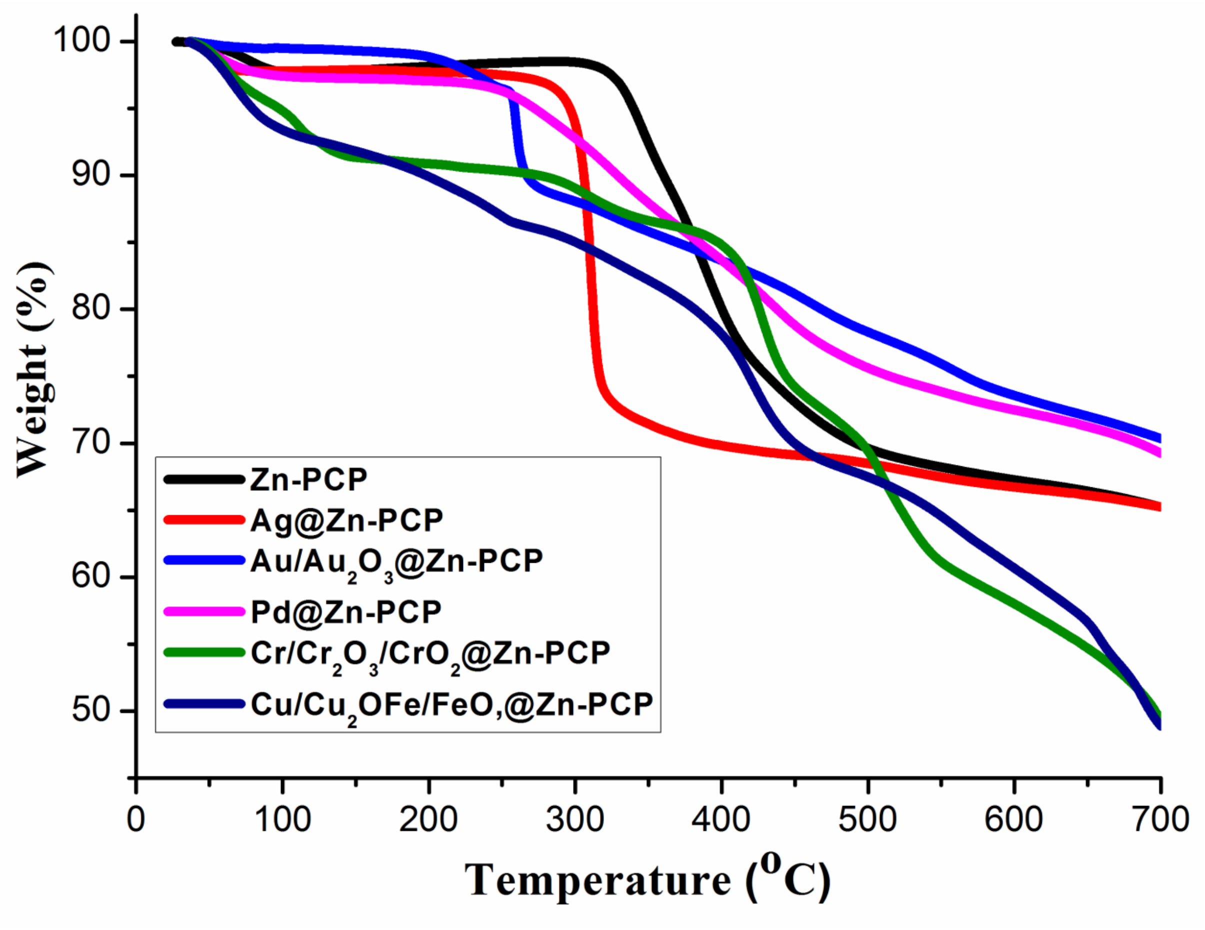

Figure S10. TGA plots of NPs integrated frameworks.

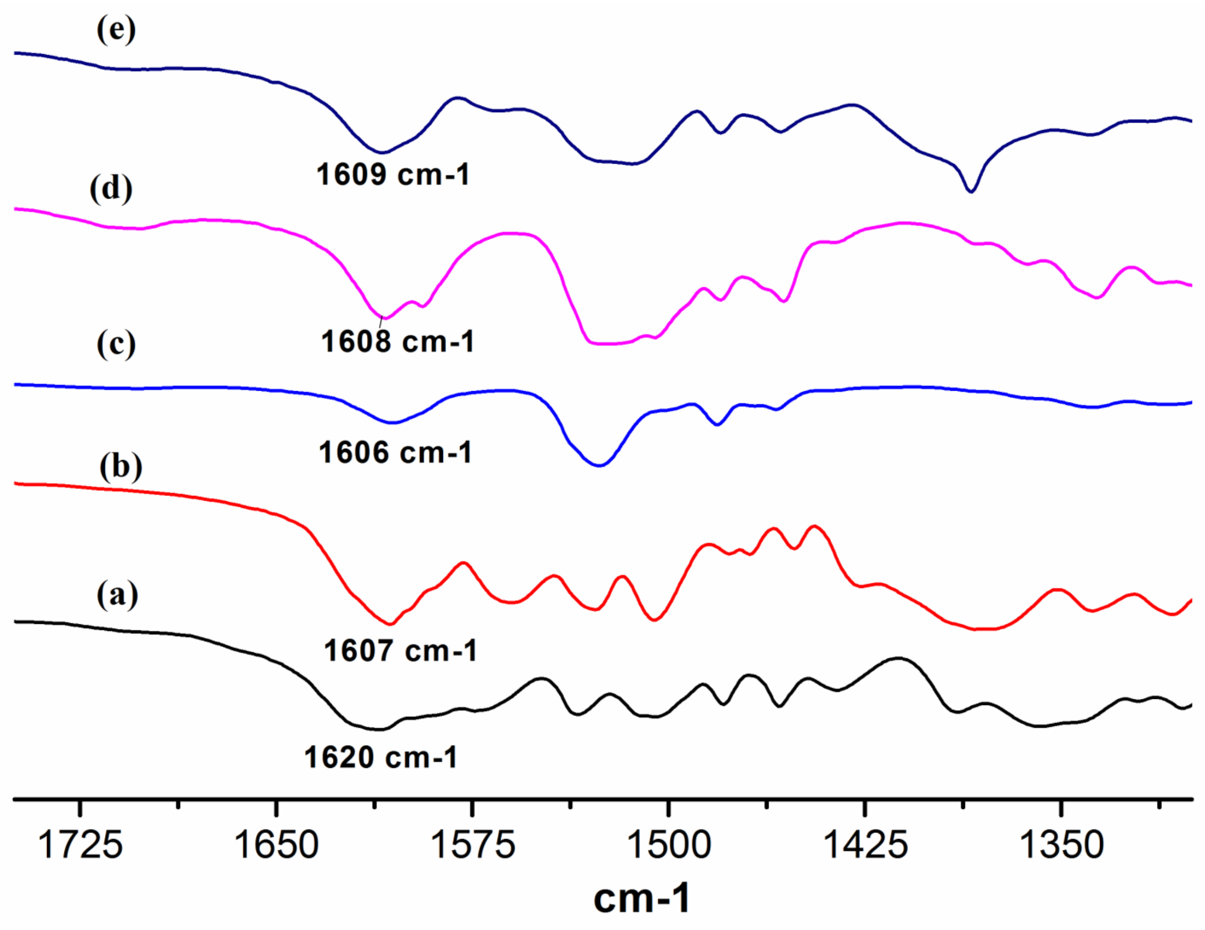

Figure S11. FTIR spectra showing C=O stretching. (a) Zn-PCP. **(b) Ag@Zn-PCP**. **(c) Au/Au_2O_3@Zn-PCP**. **(d) Pd@Zn-PCP**. **(e) Cu/Cu_2O,Fe/FeO@Zn-PCP**.

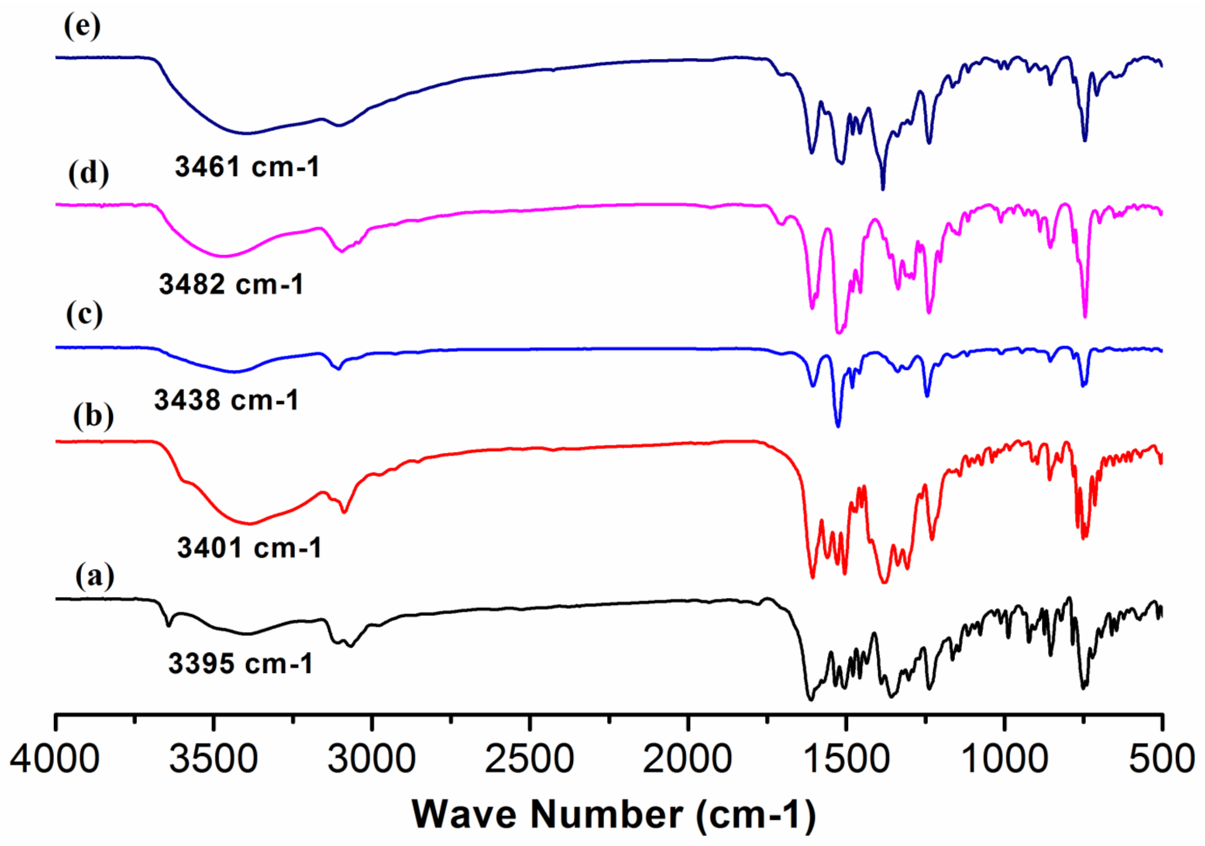

Figure S12. FTIR spectra showing O-H stretching of water solvent molecules. (a) Zn-PCP. **(b) Ag@Zn-PCP**. (c) **Au/Au_2O_3@Zn-PCP**. (d) **Pd@Zn-PCP**. (e) **Cu/Cu_2O,Fe/FeO@Zn-PCP**.

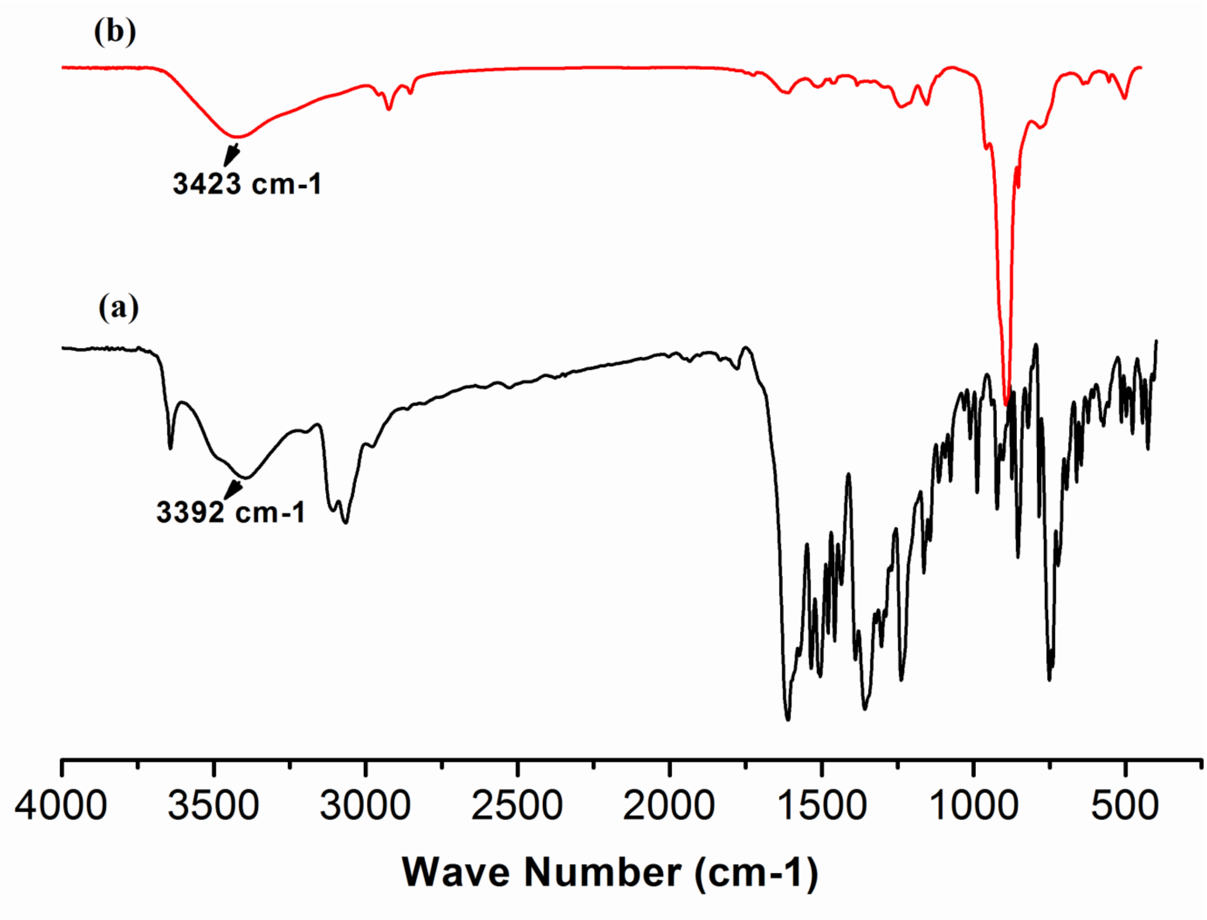

Figure S13. FTIR spectra showing narrow band of O-H^{-} stretching of Cr NPs encapsulated framework. (a) Zn-PCP. **(b) $Cr/Cr_2O_3/CrO_2$@Zn-PCP**.
